# ProtPhylo: identification of protein–phenotype and protein–protein functional associations via phylogenetic profiling

**DOI:** 10.1093/nar/gkv455

**Published:** 2015-05-08

**Authors:** Yiming Cheng, Fabiana Perocchi

**Affiliations:** 1 Gene Center, Ludwig-Maximilians-University, Munich, Bavaria 81377, Germany; 2 Institute of Human Genetics, Helmholtz Zentrum München, Neuherberg, Bavaria 85764, Germany

## Abstract

ProtPhylo is a web-based tool to identify proteins that are functionally linked to either a phenotype or a protein of interest based on co-evolution. ProtPhylo infers functional associations by comparing protein phylogenetic profiles (co-occurrence patterns of orthology relationships) for more than 9.7 million non-redundant protein sequences from all three domains of life. Users can query any of 2048 fully sequenced organisms, including 1678 bacteria, 255 eukaryotes and 115 archaea. In addition, they can tailor ProtPhylo to a particular kind of biological question by choosing among four main orthology inference methods based either on pair-wise sequence comparisons (One-way Best Hits and Best Reciprocal Hits) or clustering of orthologous proteins across multiple species (OrthoMCL and eggNOG). Next, ProtPhylo ranks phylogenetic neighbors of query proteins or phenotypic properties using the Hamming distance as a measure of similarity between pairs of phylogenetic profiles. Candidate hits can be easily and flexibly prioritized by complementary clues on subcellular localization, known protein–protein interactions, membrane spanning regions and protein domains. The resulting protein list can be quickly exported into a csv text file for further analyses. ProtPhylo is freely available at http://www.protphylo.org.

## INTRODUCTION

Advances in sequencing technologies and genome annotation tools continuously increase the repertoire of protein-coding genes in numerous organisms. The number of sequenced genomes is growing exponentially, with over 10 000 prokaryotic and eukaryotic species sequenced to date (1). However, with the exception of well-studied model organisms, the majority of species-specific protein sets remains functionally uncharacterized even though high-throughput functional annotation projects have been initialized (2). One of the most direct approaches to understand the function of a protein of interest consists of elucidating its interaction partners. Functional links can be obtained for example by experimental analysis of physical protein–protein interactions (e.g. protein complex purification) or gene–gene relationships (e.g. double mutants phenotyping and correlated gene expression). However, genome-wide surveys of functional associations remain experimentally challenging in many organisms (3).

*In silico*-based predictions of functionally linked proteins often allow inferring a function for uncharacterized components via 'guilt-by-association' with known components (4,5). One such method is based on phylogenetic profiling (6), whose predictive power increases as more sequenced genomes from diverse taxonomic groups become available (7). Phylogenetic profiling predicts functional associations on the assumption that if proteins co-occur, despite multiple evolutionary events of speciation, gene loss and lateral transfer across a large number of genomes, then they are functionally coupled. The first step involves the identification of orthologs for a protein of interest in several genomes, defining what is called a 'protein phylogenetic profile'. Next is the search for proteins, within the same genome, that show a correlated pattern of presence and absence. Many successful case studies support the application of phylogenetic profiling to identify novel components of a biological process (e.g. a biochemical pathway or a multi-subunit protein complex) (8,9), to annotate orphan proteins (10,11) and to discover proteins underlying a phenotype of interest (12,13).

Here we present ProtPhylo (www.protphylo.org), a web-based tool for prediction of protein-to-protein and phenotype-to-protein functional associations based on phylogenetic profiling. ProtPhylo achieves flexibility and state-of-the-art taxonomic and functional coverage by generating phylogenetic profiles for 9.7 million non-redundant protein sequences across 2048 organisms and by implementing four independent orthology detection algorithms. In addition, it provides an integrated framework for fast, easy and flexible prioritization of phylogenetic neighbors based on widely used tools for prediction of subcellular localization (14–18), protein domains (19), membrane spanning regions (18,20) and complementary evidence of protein–protein interactions (21).

## THE PROTPHYLO PIPELINE

### Genome selection

**Figure 1.**
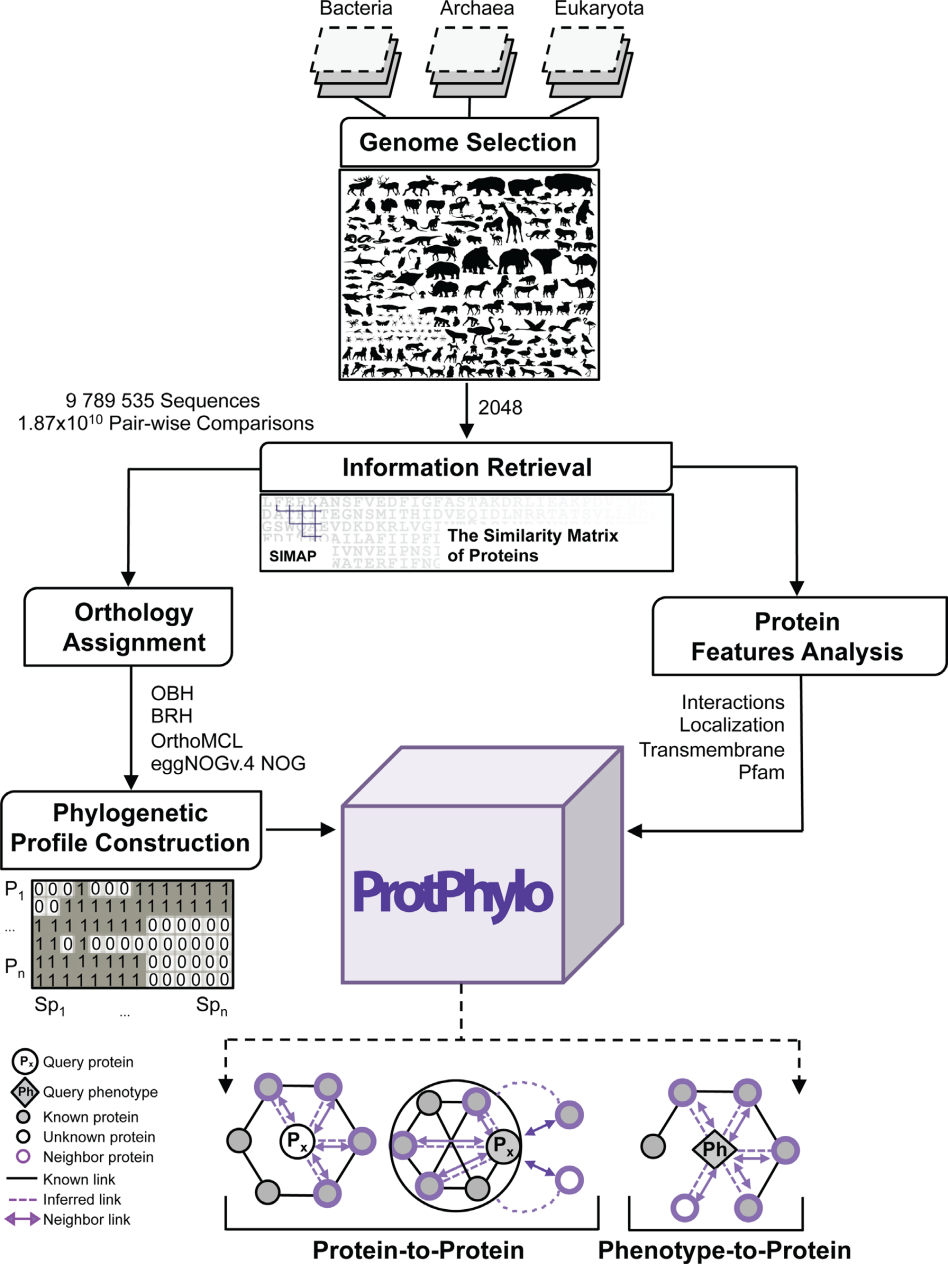
Schematic representation of the ProtPhylo Pipeline.

The first step in the ProtPhylo pipeline is the selection of relevant genomes for phylogenetic profiling (Figure 1). As in EggNOGv.4 (22), we restricted our analysis to publicly available and high-quality genome datasets as for genomic completeness, sequencing coverage and accuracy of genome annotation. While such criteria help minimizing false orthology assignment (23,24), the inclusion of species from multiple taxonomic levels at different evolutionary distances is key to maximize the resolution of coupled evolutionary patterns (7). The resulting species set covers a total of 2048 organisms, including 1678 bacteria, 115 archaea and 255 eukaryotes. The species list can be downloaded directly from the ProtPhylo web server.

### Retrieval of sequence similarity scores

For all 2048 organisms, non-redundant protein sequences were retrieved from the Similarity Matrix of Proteins (SIMAP) database (25) and filtered by sequence length (>10 amino acids) and quality (<20% non-standard amino acids). The corresponding 9 789 535 protein sequences represent a nearly even sampling of prokaryotic and eukaryotic proteomes (Table 1). Roughly, 86% of all sequences could be annotated based on protein IDs mapping between the original sequence repository (ENSEMBL, NCBI RefSeq, JGI) and Uniprot database. Sequence annotations for *Saccharomyces cerevisiae, Caenorhabditis elegans* and *Danio rerio* were based on the Saccharomyces Genome Database, Wormbase and ZFIN database, respectively. Sequence similarity scores for 1.87 x 10^10^ pair-wise comparisons were generated by the SIMAP initiative as described in EggNOGv.4 (22). Briefly, sequence alignments and similarity scores were generated with the FASTA algorithm and then recalculated using the basic local alignment search tool (BLAST) with compositional adjustment of the amino acid substitution matrix and bit score cutoff ≥50.

**Table 1.** Source and number of non-redundant sequences for 2048 organisms included in ProtPhylo as of February 2015

Kingdom (# species)	Non-redundant sequences	Database
Bacteria (1678)	5 559 635	NCBI RefSeq
Archaea (115)	256 635	NCBI RefSeq
Eukaryota (255)	3 973 265	ENSEMBL, Uniprot,
		NCBI RefSeq, JGI

### Orthology assignment and construction of phylogenetic profiles

**Figure 2.**
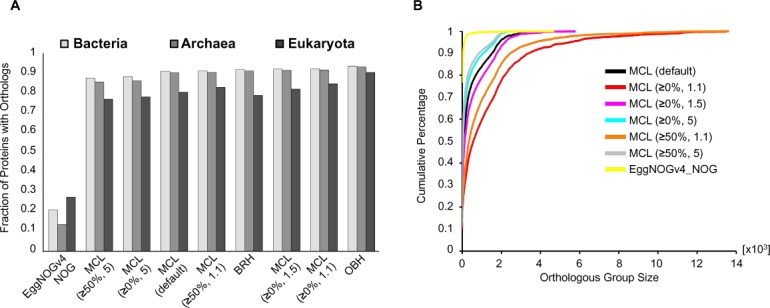
Differences in orthology prediction methods. (**A**) Percentage of proteins in each kingdom having at least one orthologous protein in any other species. (**B**) Cumulative percentage of proteins for different orthologous group size. The smaller the inflation index or the percent match length, the larger the orthologous group size. MCL (≥x, y) refers to the OrthoMCL algorithm with x representing the percent match length and y representing the inflation index.

Accurate orthology prediction is a crucial step for the construction of protein phylogenetic profiles. An overwhelming number of alternative methodologies exist for genome-wide orthology inference (26) and several attempts have already been made to compare their relative performance (27,28). However, it remains challenging to draw a conclusion on which method is the best. Instead, the choice of an orthology detection method over others largely depends on the kind of functional conservation being predicted (e.g. co-expression, molecular function, involvement in similar pathways, protein–protein interaction, etc.) as well as on the number, diversity and evolutionary distances of the species being compared (26–28). Currently, ProtPhylo implements four commonly used, BLAST-based algorithms for orthology assignments (Figure 2A): One-way Best-Hits (OBH), Best-Reciprocal-Hits (BRH) (29), OrthoMCL (30) and eggNOGv.4 (22). OBH and BRH rely on pair-wise sequence comparisons and determine orthology using a simple BLAST cut-off criterion, (*E*-value < 10^−5^). Instead, OrthoMCL and eggNOGv.4 extend the sequence similarity search over multiple proteomes at once to generate groups of orthologous proteins. Both, OrthoMCL and eggNOGv.4 algorithms, derive orthologous groups based on BRH (*E*-value < 10^−^^5^) as their first step but apply different clustering techniques to assemble protein groups from multiple species, Markov Cluster (MCL) (30) and triangular linkage (31), respectively. In addition to the default parameters used in OrthoMCL, ProtPhylo derives orthologous groups also based on different settings for percent match length (≥0 or ≥50%) and inflation index (1.1, 1.5 or 5). The first refers to the percentage of positive-scoring matches of the high-scoring pairs and the second corresponds to the parameter used by MCL to define the tightness of orthologous groups. Here, the higher the inflation indexes, the tighter the size of the orthologous groups (Figure 2B).

Next, for all 9.7 million proteins we generated phylogenetic profiles across 2048 organisms using the abovementioned orthology inference methods. Here, the phylogenetic profile of a protein or a phenotype is represented by a binary string with *n* entries, where *n* corresponds to the size of the species set and the entry indicates the presence (1) or absence (0) of an ortholog or a similar phenotype across species.

### Retrieval of protein features

ProtPhylo includes functional annotations for all 9.7 million proteins based on evidence of subcellular localization (14–18), presence of transmembrane helices (18,20), protein domain families (19) and complementary evidence of protein–protein interactions (21). Subcellular localization is either predicted by applying sequence-based computational strategies, as in TargetP 1.1 (15), MitoProt II (16), and LocTree3 (14), or retrieved by Uniprot database. Briefly, TargetP 1.1 uses N-terminal sequence information to discriminate between proteins targeted to the mitochondrion, the chloroplast, the secretory pathway and ‘others’. MitoProt II computes the probability (*MitoProt II Score*) that a protein has a mitochondrial-targeting sequence. Prediction of mitochondrial localization for *Homo sapiens* and *Mus musculus* is also retrieved from MitoCarta (17). LocTree3 predicts protein localization to at least twelve of the major subcellular locations of prokaryotic and eukaryotic cells (nucleus, cytoplasm, mitochondrion, plasma membrane, Golgi apparatus, endoplasmic reticulum, vacuole, peroxisome, plastid, chloroplast, extracellular region and fimbrium). Currently, LocTree3 predictions are available for ∼1500 species in ProtPhylo (68% of protein sequences). Protein transmembrane helices are predicted by using TMHMM v2.0 algorithm (20) as well as annotated from Uniprot database based on experimental and computational evidence. Protein domain families are predicted by hmmscan program against Pfam-A domains (Pfam 27.0) (19).

## PROTPHYLO WEB SERVER IMPLEMENTATION

### Input

Users can search for proteins, within a query organism, that co-evolve with either a phenotype (*Phenotype Phylogenetic Profiling*), for example ion uptake, thermogenesis, or multicellularity, or a protein of interest (*Protein Phylogenetic Profiling*) and are therefore likely to be functionally associated. *Phenotype Phylogenetic Profiling* can only be applied when users have *a priori* knowledge of the presence and/or the absence of the phenotype across any of the 2048 organisms in ProtPhylo (*Species WITH and/or* Species WITHOUT the Phenotype of Interest). In both search options, users can select among four main orthology methods (OBH, BRH, OrthoMCL and eggNOGv.4), as well as five additional OrthoMCL settings (OBH is set as the default orthology method). ProtPhylo uses all 2048 organisms to generate a protein phylogenetic profile, while the set of species used to generate a phenotype phylogenetic profile can be defined by the user.

### Output

ProtPhylo compares the query phylogenetic profiles (protein or phenotype) to the profiles of all other proteins within the query organism and ranks proteins based on their similarity scores, from the smallest (closest phylogenetic neighbor) to the largest. Here, we use the Hamming distance (HD) to quantify the similarity between pairs of phylogenetic profiles (6). This corresponds to the number of positions whereby the two binary vectors have different entries. In total, ProtPhylo takes ∼2 s of calculation time for each query, when default settings are used. In addition to the HD, each row of the output list (phylogenetic neighbor) contains information on protein IDs and names from relevant protein repositories. Further protein details can be found through hyperlinks (*Source Protein ID*). When selecting OrthoMCL or eggNOGv.4 as orthology detection methods, users can also retrieve the list of orthologs for each ranked protein (*Orthologs*). For OBH and BRH, the retrieval of orthologs is available for the following query organisms*: H. sapiens, M. musculus, S. cerevisiae, C. elegans, D. melanogaster* and *D. rerio*. The output of a *Protein Phylogenetic Profiling* search includes two additional calculations: the *HD Percentile* and the *Reciprocal HD Percentile*. The former refers to the percentage of proteins within the query proteome that have equal or lower HD than the phylogenetic neighbor HD, while the latter is calculated when clicking the magnifier icon and reflects the HD percentile of the user-defined query protein if the phylogenetic neighbor is used as a query. By default, ProtPhylo reports a protein list with less than or equal to the fifth percentile. The output list can be further prioritized directly in ProtPhylo based on five complementary filtering criteria: cut-off HD values and percentile; combined (*And*) or stand-alone (*Or*) evidence of subcellular localization; presence (*>0*) or absence (*=0*) of transmembrane helices; presence of conserved Pfam domains (by Pfam ID or name); keywords (gene symbol, protein IDs and names); confidence score for functional associations predicted by STRING (21), (*STRING score*).

### Phenotype-to-protein: the MCU case study

**Figure 3.**
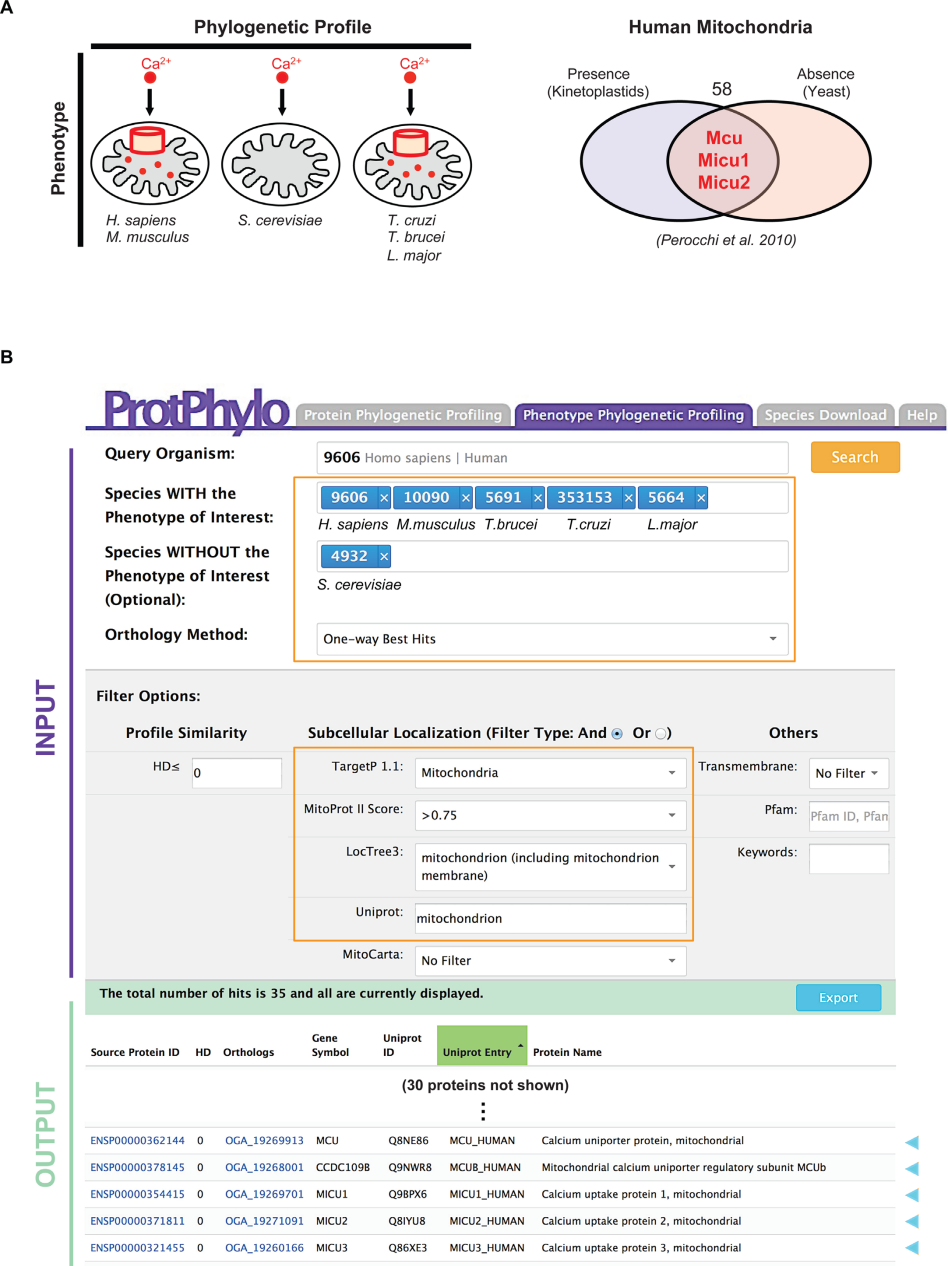
Example of phenotype-to-protein functional associations predicted by *Phenotype Phylogenetic Profiling* in ProtPhylo. (**A**) Mitochondria calcium uptake (Phenotype) is common to vertebrate and protozoa, yet not measurable in *Saccharomyces cerevisiae* (Phylogenetic Profile). Human proteins of the mitochondrial calcium uptake channel were predicted by looking for mitochondria-localized proteins that have the same phylogenetic profile of the calcium uptake phenotype (12) and have predicted transmembrane domains (13). (**B**) The ProtPhylo web interface query for the phenotype described in (A). Known components of the human (*query organism*) mitochondrial calcium uniporter are found within the 35 phylogenetic neighbors predicted by ProtPhylo.

As an illustrative example of *Phenotype Phylogenetic Profiling*, we used ProtPhylo web interface to predict human proteins that co-evolve with the ability of mitochondria to uptake calcium via a uniport mechanism (32,33). The underlying machinery, called ‘the mitochondrial calcium uniporter’ (MCU), consists of a highly selective calcium channel with unique biophysical properties, being high-capacity, dependent on membrane potential, calcium-selective even at low concentration of free cytosolic calcium and sensitive to nanomolar concentrations of ruthenium derivatives (34). Despite intense efforts dating back to over 60 years ago, the molecular nature of MCU has evaded traditional biochemical strategies as well as genome-wide RNAi screens. Recently, this mystery was uncovered by searching for human mitochondrial proteins that are conserved in vertebrates and kinetoplastids but not in yeast, which is unable to perform calcium uptake (12), (Figure 3A). Here, phylogenetic profiling was applied to predict phenotype-to-protein functional associations, leading to the discovery of uniporter's regulatory and structural subunits, Micu1-2 and Mcu, respectively (12,13). As shown in Figure 3B, ProtPhylo predicts 35 human proteins as having the same phylogenetic profile of the phenotype of interest (HD = 0) across the six taxa and being localized to mitochondria (combined evidence from MitoProt II score, TargetP 1.1, Loctree3, and Uniprot). Candidate proteins include Micu1-2 and Mcu, as well as other components of the uniporter protein complex such as Mcub (35).

### Protein-to-protein: the Stim1 case study

**Figure 4.**
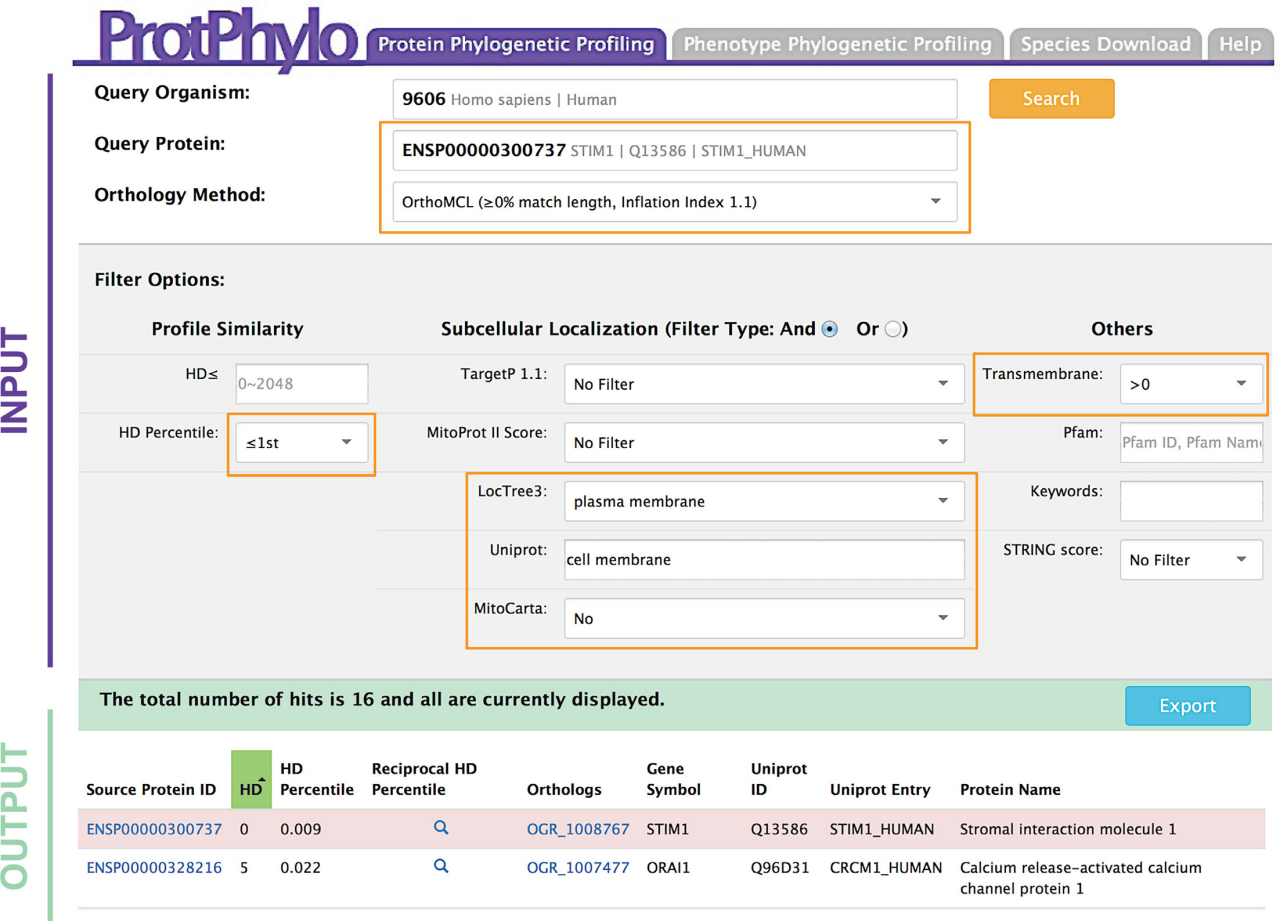
Example of *Protein Phylogenetic Profiling*. The ProtPhylo web interface query used to predict human proteins (*query organism*) functionally associated with Stim1 (*query protein*) is shown. Here, OrthoMCL (≥0% match length, inflation index 1.1) is used as orthology detection method. Known components of the CRAC channel represent the top phylogenetic neighbors (smallest HD).

As an illustrative example of *Protein Phylogenetic Profiling*, we used ProtPhylo to predict proteins that are functionally linked to Stim1, stromal interaction molecule 1. Stim1 is a key signaling protein regulating the influx of calcium through the plasma membrane in response to InsP3-induced depletion of the endoplasmic reticulum calcium pool (36). Calcium entry through the plasma membrane occurs through Calcium Release-Activated Calcium (CRAC) channels whose molecular identity remained unknown till 2006. After the discovery of Stim1 in 2005 by genome-wide RNAi screening efforts (37,38), it followed the identification of the CRAC channel subunit, Orai1, through three other genome-wide RNAi screens (39–41). Here, we asked whether ProtPhylo could predict the functional association between Stim1 and Orai1 solely based on phylogenetic profiling and protein-feature analysis. As shown in Figure 4, we searched for human proteins that co-occur with Stim1 across all 2048 organisms (*≤1st HD percentile*), localize to the plasma membrane (*LocTree3, Uniprot*) but not to the mitochondria (*MitoCarta*) and have at least one transmembrane domain. As a result, ProtPhylo identifies 16 proteins matching the selected criteria, with Orai1 being the top phylogenetic neighbor of Stim1 (HD = 5).

## DISCUSSION

**Figure 5.**
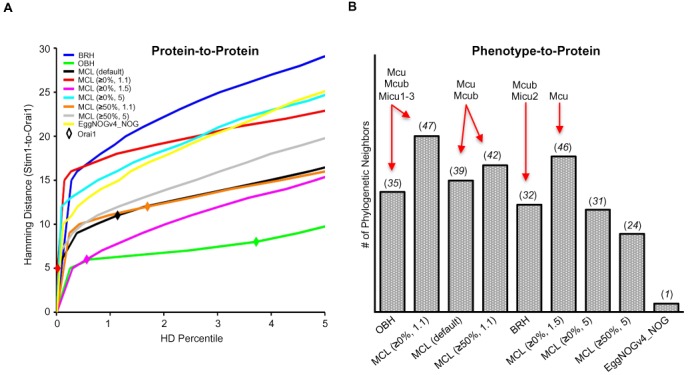
Effect of different orthology detection methods on phylogenetic profiling. (**A**) Hamming distance between human Stim1 (query protein) and Orai1 (phylogenetic neighbor) when using different orthology methods and parameters. The strongest functional association (lower HD and HD Percentile) can be predicted using OrthoMCL (≥0% match length, inflation index 1.1). (**B**) Number of phylogenetic neighbor proteins predicted to co-evolve with the calcium uptake phenotype (HD = 0) based on different orthology detection methods. The presence of known components of the mitochondrial calcium uptake channel is shown.

ProtPhylo aims to provide a fast, flexible and user-friendly tool to assist biologists seeking functional clues for a protein or a phenotype of interest. Several features distinguish ProtPhylo from other web servers (21,30,42–43). First, it implements different orthology detection methods to generate phylogenetic profiles. Second, ProtPhylo can operate on two types of inputs, protein-based and phenotype-based phylogenetic profiles. Third, it covers three domains of life and retrieves functional associations for proteins from any of 2048 organisms. To emphasize its suitability as a discovery tool, we validated its performance with datasets of known human protein complexes (CORUM, (44)), cellular components from the Gene Ontology (GO) database (45) and metabolic and signaling pathways from KEGG (46). Overall, we find that ProtPhylo reaches the highest protein pairs recall rate when applied to CORUM dataset of 1736 manually curated human protein complexes (data not shown). Varying the orthology method for phylogenetic analysis in ProtPhylo shows little effect on protein pairs recall rates. However, when combining all orthology methods, the recall rate increases for all three datasets, indicating that different orthology methods recall a different subset of true positive interactions, as also shown in Figure 5. This highlights the value of including more than one orthology method in ProtPhylo, a unique feature that distinguishes ProtPhylo from other web servers (21,30,42–43). Therefore, it could be advantageous to run ProtPhylo with different methods or use them in combination to increase the rate of true positive predictions, while using other filtering options available in ProtPhylo to decrease the rate of false positives. In summary, ProtPhylo web server offers users the possibility to narrow down the number of testable hypotheses through the extension of phylogenetic profiling and comparative biology analyses to an ever growing sequence space.
